# Interaction of Sp1 and Setd8 promotes vascular smooth muscle cells apoptosis by activating Mark4 in vascular calcification

**DOI:** 10.18632/aging.205492

**Published:** 2024-02-01

**Authors:** Yun Li, Meijuan Cheng, Jingjing Jin, Dongxue Zhang, Shenglei Zhang, Yaling Bai, Jinsheng Xu

**Affiliations:** 1 Department of Nephrology, The Fourth Hospital of Hebei Medical University, Shijiazhuang 050011, People’s Republic of China; 2 Hebei Clinical Research Center for Chronic Kidney Disease, Hebei Key Laboratory of Vascular Calcification in Kidney Disease, Shijiazhuang, People’s Republic of China

**Keywords:** vascular calcification, apoptosis, Mark4, Sp1, Setd8

## Abstract

Vascular calcification (VC) is directly related to high mortality in chronic kidney disease (CKD), and cellular apoptosis of vascular smooth muscle cells (VSMCs) is a crucial process in the initiation of VC. Microtubule affinity-regulating kinase 4 (Mark4), known as a serine/threonine protein kinase, can induce cell apoptosis and autophagy by modulating Akt phosphorylation. However, the potential functions and molecular mechanisms of Mark4 in VSMCs apoptosis and calcification need to be further explored. Initially, our data indicated that the mRNA expression of Mark4 was prominently elevated in high phosphorus-stimulated human VSMCs compared with the other members in Marks. Consistently, Mark4 expression was found to be significantly increased in the calcified arteries of both CKD patients and rats. *In vitro*, silencing Mark4 suppressed apoptosis-specific marker expression by promoting Akt phosphorylation, finally attenuating VSMCs calcification induced by high phosphate. Mechanically, the transcription factor Sp1 was enriched in the Mark4 promoter region and modulated Mark4 transcription. Moreover, SET domain-containing protein 8 (Setd8) was proved to interact with Sp1 and jointly participated in the transcriptional regulation of Mark4. Finally, rescue experiments revealed that Setd8 contributed to VSMCs apoptosis and calcification by modulating Mark4 expression. In conclusion, these findings reveal that Mark4 is transcriptionally activated by Sp1, which is interacted with Setd8, to promote VSMCs calcification through Akt-mediated antiapoptotic effects, suggesting that Mark4 represents a potent and promising therapeutic target for VC in CKD.

## INTRODUCTION

Vascular calcification (VC), especially in the tunica media, is highly prevalent in chronic kidney disease (CKD) [1, 2] and may result in adverse cardiovascular events [3]. Once cardiovascular diseases occur, the cost of treatment and mortality are both increased, but limited prevention and treatment options are available for VC. Consequently, identifying the potential mechanisms of VC is crucial to reveal the pathological progression and explore effective therapeutic targets. Accumulated evidence suggests that vascular smooth muscle cells (VSMCs) apoptosis is an important regulatory process in VC, which initiates and promotes mineral deposition primarily through exposing phosphatidylserine and releasing apoptotic bodies [4]. The intrinsic apoptosis pathway in VSMCs can be triggered by caspase activation and mitochondrial changes, which are regulated by decreased expression of the antiapoptotic protein B-cell lymphoma-2 (Bcl-2) and increased expression of the proapoptotic protein BCL2-associated X (Bax) in high phosphate conditions [5, 6].

Mark4, ubiquitously expressed in mammalian cells, is a serine/threonine protein kinase and comprised of a polypeptide with a length of 752 amino acid residues [7]. The Mark4 protein is highly evolutionarily conserved, and its main biological function is to phosphorylate microtubule-associated proteins, thereby increasing microtubule dynamics and regulating cell proliferation, division, and programmed death [8, 9]. Previous studies have shown that Mark4 co-localized with centrosomes and microtubules in eukaryotic cells [10]. Depletion of Mark4 in fibroblasts and glioma cells altered the centrosome cycle and arrested cells in G1 phase, thereby affecting their proliferation [10, 11]. In comparison, Mark4 upregulation in neurons in mice with focal cerebra ischemia led to immediate cell death due to microtubule structure disassembly [12]. In addition to microtubule, Mark4 can promote VSMCs proliferation and trigger adipocytes autophagy or apoptosis by modulating diverse signaling pathways, including the Hippo/YAP/TAZ [13, 14], PI3K/Akt [15], and JNK1 signaling pathways [16], which in turn participated in the regulation of atherosclerosis. Nevertheless, the potential role of Mark4 in VSMCs apoptosis has not been studied.

Specific protein 1 (Sp1), as a eukaryotic transcriptional activator, was the first identified member of the Sp/KLF transcription factor family [17]. Sp1 C-terminal-specific zinc fingers can bind to promoters of GC box-rich target genes and regulate their transcription in various disease states [18–21]. Given that Sp1 acts as an essential transcription factor for the regulation of multiple genes involved in VSMCs proliferation and migration, vascular inflammation, cholesterol homeostasis and triglyceride metabolism, it is reasonable to conclude that Sp1 plays a key role in cardiovascular-related diseases [22]. Additionally, Sp1 has been reported to transcriptionally regulate several proapoptotic factors, such as tumor necrosis factor-related apoptosis-inducing ligand, myeloid cell leukemia 1, and Fas ligand, thereby affecting cell resistance or sensitivity to apoptosis [23]. Furthermore, Sp1 can activate the expression of serine/threonine kinase 39 at the transcriptional level, while serine/threonine kinase 39 and Mark4 belong to the same family [24]. However, whether Sp1 is implicated in modulating Mark4 expression in VSMCs apoptosis needs to be further explored.

SET domain-containing protein 8 (Setd8) is the only nucleosome-specific methyltransferase known to modulate the methylation levels of H4K20 [25]. Setd8-mediated H4K20me1 can regulate chromatin structure and accessibility, thus cooperating with transcription factors to manage target gene expression [26–28]. The results from our previous study demonstrated that Setd8 suppression contributed to high phosphate-induced VSMCs apoptosis and calcification [29]. Whereas, the underlying molecular mechanism remains poorly understood. Given this background, in this study, we performed further investigations and revealed that Mark4 expression was significantly up-regulated in calcification models. Silencing Mark4 promoted Akt-related antiapoptotic effects and inhibited VC. Mechanistically, Sp1 was found to function as a transcription factor cooperated with Setd8 to positively regulate Mark4 expression through modulating its transcriptional activity, thus promoting the occurrence and progression of VC. Overall, this study may provide novel mechanistic insights into preventive and therapeutic strategies for VC in patients with CKD.

## RESULTS

### Mark4 was upregulated and associated with VC in patients with CKD

**Figure 1:**
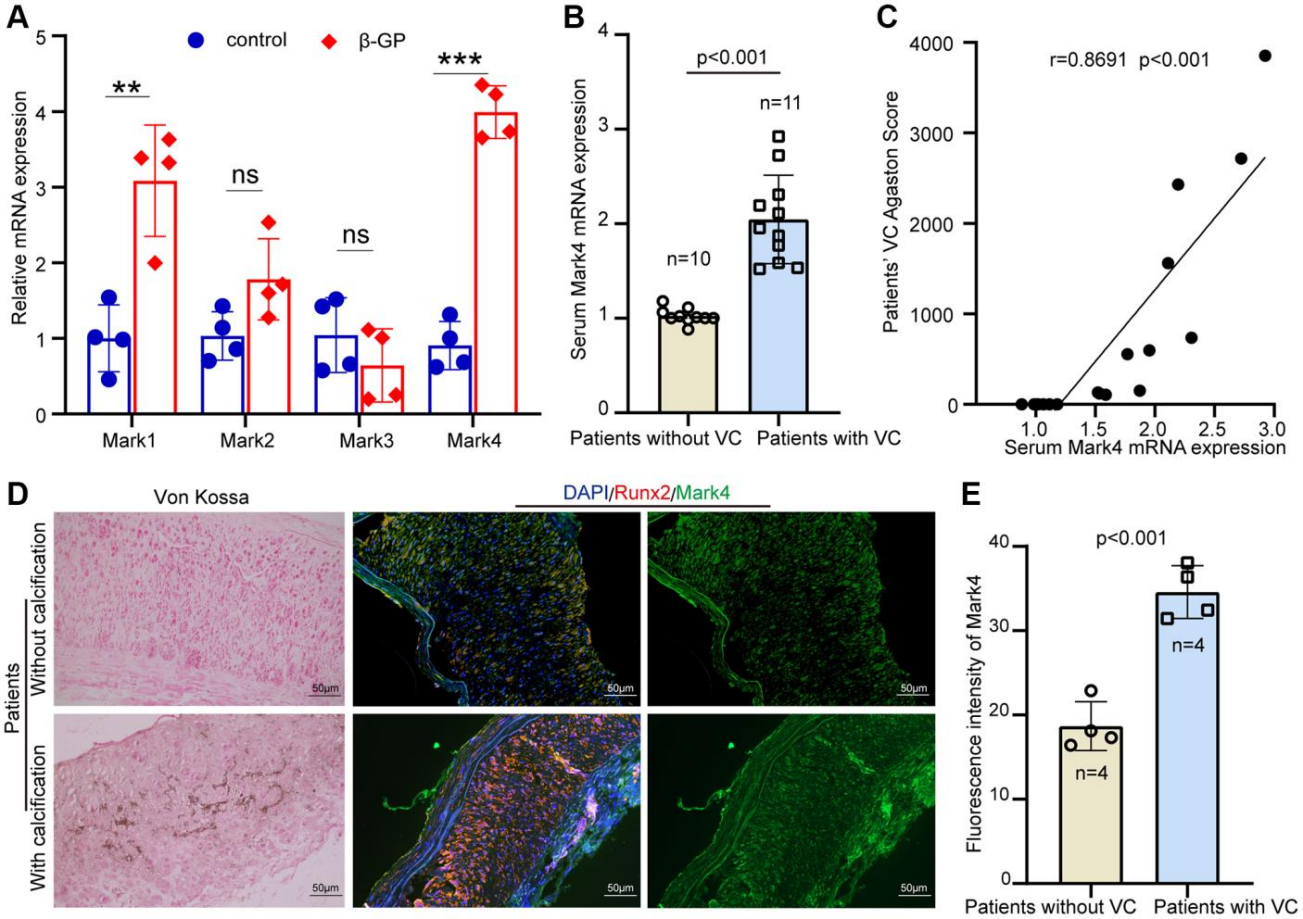
**High level of Mark4 expression was associated with increased risk of vascular calcification.** (**A**) The RT–qPCR showed expression of Marks 1- 4 in HASMCs with high phosphate (*n* = 4 per group). Error bar represent four independent experiments. ^**^Means *p* < 0.01, ^***^p < 0.001 vs. control group. (**B**) Mark4 mRNA level in serum from patients from CKD with (*n* = 11) or without VC (*n* = 10). (**C**) Correlation between the Mark4 mRNA level and VC scores in patients with CKD (*n* = 21, the Pearson’s correlation coefficient r value and the *p*-value are shown). (**D**) Von Kossa assay and IF staining for Mark4 in radial arteries sections from patients with CKD (*n* = 4 per group). Scale bars: 50 μm. (**E**) The bars showing fluorescence intensity of Mark4 in arteries between patients from CKD with or without VC (*n* = 4 per group). Error bar represent four independent experiments.

First, the expression levels of Mark mRNA (Mark1-4) in human VSMCs mediated by β-GP were gauged by Realtime-quantitative Polymerase Chain Reaction (RT-qPCR). As shown in Figure 1A, Mark4 mRNA expression was the most significantly elevated among the four Mark family members. To preliminarily investigate the relationship between Mark4 and VC, Mark4 expression was measured in serum samples from 21 CKD individuals with or without VC. The characteristics of the CKD patients were shown in Table 1. Patients with VC presented dramatically higher Mark4 levels than controls without VC (Figure 1B). Interestingly, Mark4 expression was positively correlated with the increased severity of VC (*P* < 0.001; Figure 1C). To further clarify whether Mark4 took a crucial part in VC, we evaluated Mark4 and Runx2 (osteogenic marker) expression in calcified radial arteries from patients undergoing an arteriovenostomy before hemodialysis. Von Kossa assays were employed to identify matrix mineral deposition, and immunofluorescence (IF) staining for Mark4 and Runx2 was performed in arterial tunica media. The arteries with calcification arteries exhibited significantly elevated Mark4 (green fluorescence) and Runx2 (red fluorescence) staining intensity compared with those without calcification (Figure 1D, 1E). Collectively, these data suggested that Mark4 might be involved in VC progression among patients with CKD.

**Table 1:** Basal characteristics in patients with CKD with or without calcification.

	**Non-VC (*n* = 10)**	**VC (*n* = 11)**	***P*-value**
**Demographic characteristics**			
Age, years	45.4 ± 17.115	54.92 ± 13.574	0.161
Sex			0.439
Male	5 (50%)	7 (63.6%)	
Female	5 (50%)	4 (36.4%)	
Coronary disease	1 (10%)	3 (27.2%)	0.375
BMI, kg/m²	21.010 ± 5.561	23.296 ± 3.635	0.26
Dialysis ages	102.58 ± 70.204	87.854 ± 55.465	0.588
**Plasma biochemical characteristics**			
Mark4, expression	1.026 ± 0.079	2.045 ± 0.468	<0.001
Hemoglobin, g/L	117.4 ± 9.454	113.75 ± 11.92	0.869
ALB, g/L	40.8 ± 3.2218	41.267 ± 4.469	0.448
ALP, g/L	103.83 ± 39.4318	132.458 ± 51.780	0.262
Ferritin, ug/L	214.75 (93.3-324.85)	341.908 (69.75-502.4)	0.291
TS, %	21.87 (15.175-22.175)	21.25 (15.625-25.350)	0.391
Pi, mmol/L	1.839 ± 0.443	1.473 ± 0.569	0.08
Ca, mmol/L	2.151 (2.115-2.33)	2.3133 (2.112-2.447)	0.488
iPTH, pg/ml	201.01 ± 99.807	220.29 ± 137.767	1
25-OH VD, ng/ml	13.738(9.5125-16.355)	21.0583(13.7375-32.205)	0.114
K, mmol/L	5.15 ± 0.635	4.667 ± 0.600	0.075
CO_2_CP, mmol/L	23.2 ± 2.341	22.95 ± 2.109	0.692
CRP, mg/L	4.865(1.8875-8.305)	5.6608 (1.7425-4.873)	0.947
VC Angaston score	0	1178.55(129-1778.75)	<0.001

Values are expressed as mean ± SD or median (25th to 75th quartiles) for continuous variables and *n* (%) for categorical variables, respectively. Statistical significance was assessed using Student’s *t*-test (all characteristics except sex) or nonparametric Mann Whitney *U*-test (sex). Abbreviations: BMI: body mass index; K: potassium; Ca: calcium; Pi: phosphate; ALB: albumin; ALP: alkaline phosphatase; TS: Transferrin saturation degree; iPTH: Intact parathyroid hormone; 25-OH VD: 25-Hydroxy Vitamin D; CRP: C-reaction protein; CO_2_CP: carbon dioxide combining power.

### Mark4 and apoptotic factors were increased in the aortas of calcified rats and high phosphorus–induced VSMCs

**Figure 2:**
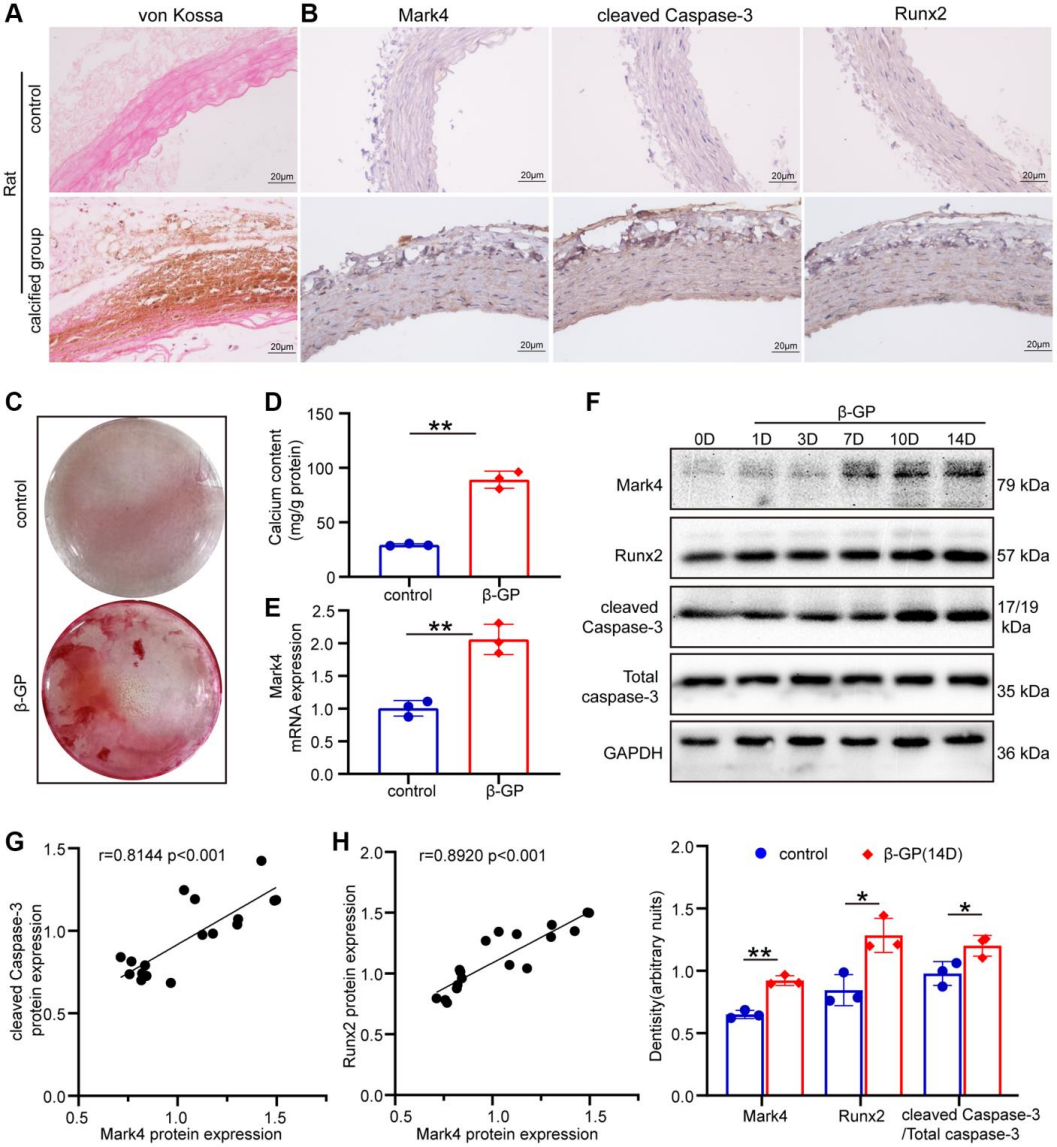
**Mark4 was upregulated and associated with apoptosis in the aortas of calcified rats and β-GP-cultured VSMCs.** (**A**) Von Kossa staining in the aortas of control rats and calcified rats. (**B**) Immunohistochemistry of Mark4, Runx2 and cleaved Caspase-3 expression in the aortas of control and calcified rats (*n* = 3 per group). Scale bars: 20 μm. (**C**) Alizarin Red S staining of VSMCs cultured with 10 mM β-glycerophosphate (β-GP) medium for 14 days. (**D**) Quantitative analysis of the calcium content in VSMCs after cultured with β-GP medium for 14 days compared with 0 days. ^**^Means *p* < 0.01. (**E**) RT–qPCR analysis of Mark4 mRNA expression in VSMCs at 14 days after β-GP induction compared with 0 days. Error bar represent three independent experiments. ^**^Means *p* < 0.01. (**F**) Western blot and statistical analyses of Mark4, Runx2 and cleaved Caspase-3 expression in VSMCs treated with β-GP for different days. Error bar represents three independent experiments. ^*^Means *p* < 0.05, ^**^p < 0.01 vs. control group. (**G**) Correlation analysis between the protein expression of Mark4 and cleaved Caspase-3. (**H**) Correlation analysis between the protein expression of Mark4 and osteogenic marker Runx2 in β-GP-induced VSMCs.

As previous evidence showed, Mark4 regulated the expression of apoptosis-related genes [16], and cleaved Caspase-3 is a molecular marker of cells undergoing apoptosis [3, 30]. Therefore, we first detected cleaved Caspase-3 and Mark4 expression in the calcified aortas of CKD rats (Figure 2A). As shown in Figure 2B, cleaved Caspase-3 and Mark4 levels were distinctly increased in the calcified arteries compared with those in the control group, accompanied by the upregulated expression of Runx2. *In vitro* cell experiments, we further corroborated that high-phosphorus medium configurated by 10 mM β-glycerophosphate (β-GP) successfully induced VSMCs calcification, evidenced by elevated red calcium nodes and the quantitation of calcium content (Figure 2C, 2D); additionally, Mark4 mRNA levels were markedly increased (Figure 2E). Similarly, the protein levels of Mark4 in VSMCs treated with high β-GP gradually increased in a time-dependent manner, parallel with the upregulation of cleaved Caspase-3 and Runx2 (Figure 2F). Moreover, correlation analysis revealed that Mark4 positively correlated with cleaved Caspase-3 and Runx2 expression in VSMCs incubated with high phosphorus (Figure 2G, 2H). Taken together, these observations indicated that Mark4 might participate in VSMCs apoptosis and calcification.

### Mark4 promoted calcification probably via inhibiting the antiapoptotic effect of the Akt signal

**Figure 3:**
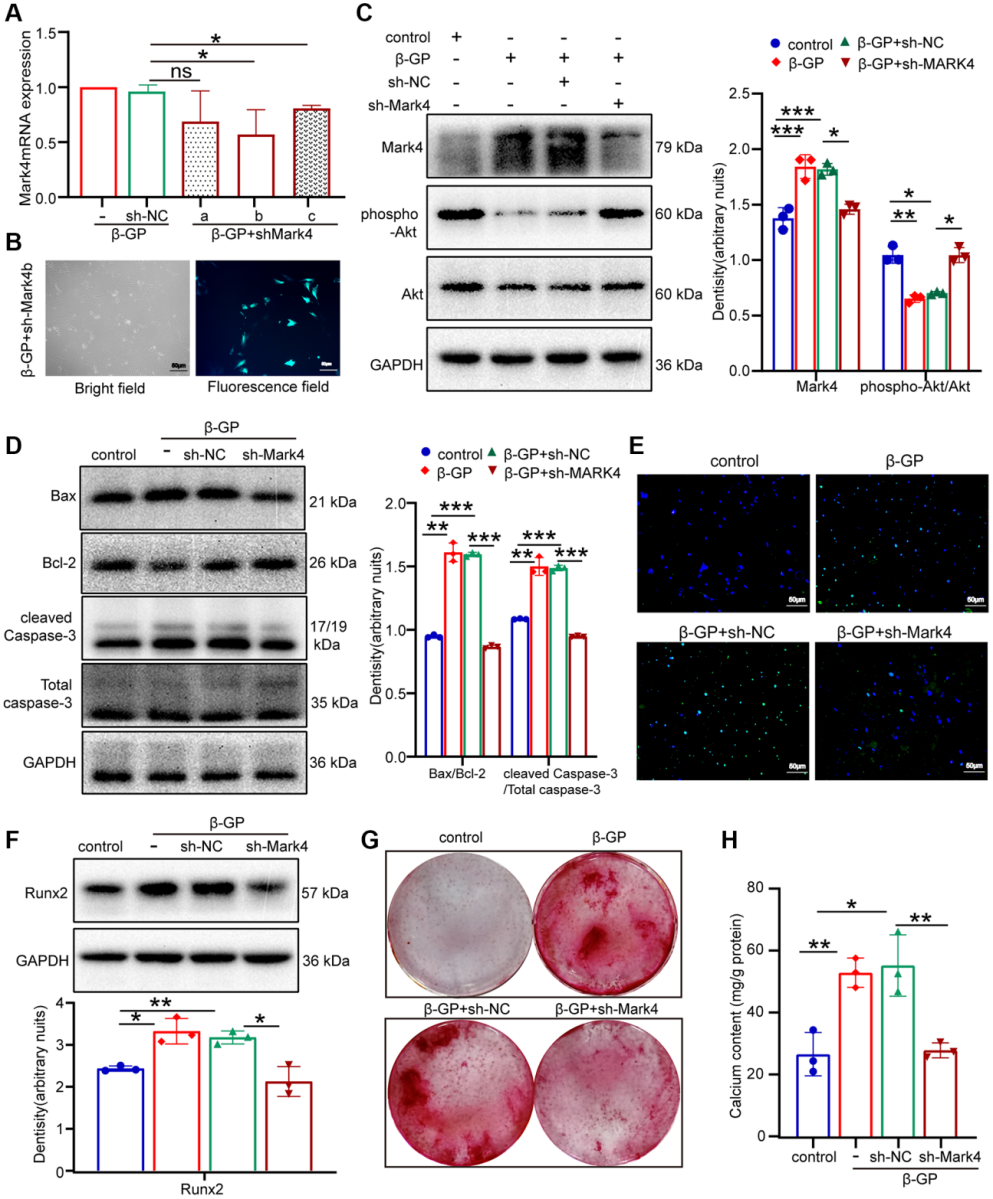
**Mark4 knockdown inhibited β-GP-induced VSMCs apoptosis and calcification.** VSMCs were infected with lentiviral shMark4 (sh-Mark4) or sh-negative control (sh-NC) under high phosphate conditions for 72 h. (**A**) The RT–qPCR showed transfection efficiency of sh-Mark4a-c in β-GP mediated VSMCs. ^*^Means *p* < 0.05 vs β-GP+ sh-NC group. (**B**) The inverted fluorescence microscopy observation of sh-Mark4b transfection efficiency in β-GP-treated VSMCs. (**C**, **D**) Western blot and quantitative densitometry analysis of Mark4, Akt and apoptosis-related proteins in VSMCs with knockdown of Mark4. (**E**) Apoptosis of sh-Mark4 transfected VSMCs cultured with β-GP medium was assessed by TUNEL staining (green). Nuclei were counterstained with DAPI (blue). (**F**) Western blot and quantitative densitometry analysis of Runx2 in VSMCs with knockdown of Mark4. (**G**) Alizarin Red S staining and (**H**) calcium content in VSMCs infected with sh-Mark4. ^*^Means *p* < 0.05, ^**^p < 0.01, ^***^p < 0.001 vs. control, ^*^p < 0.05, ^**^p < 0.01, ^***^p < 0.001 vs. β-GP+ sh-NC. Error bar represents three independent experiments.

To probe whether Mark4 played a critical role in high phosphate-mediated Akt phosphorylation and the expression of apoptotic protein, including Bax, Bcl-2, and cleaved Caspase-3 in VSMCs, three independent shRNAs against Mark4 (sh-Mark4a-c) were adopted. The efficiency was validated at the mRNA level, and sh-Mark4b was chosen for the subsequent experiments (Figure 3A). After transfection with sh-Mark4b successfully, VSMCs showed bright green fluorescence with a transfection efficiency approaching 50% (Figure 3B). The data in Figure 3C, 3D showed that Mark4 knockdown reversed the effect of high phosphate on the inhibition in Akt phosphorylation and reduced Bax/Bcl-2 and cleaved Caspase-3 levels, thus alleviating VSMCs apoptosis. Similarly, the TUNEL assay showed that Mark4 deficiency mitigated VSMCs apoptosis (Figure 3E). Meanwhile, Mark4 silencing suppressed the expression of the osteogenic marker Runx2 and attenuated calcium nodule formation and calcium deposition, as determined by Western blot, alizarin red staining and calcium assay, respectively (Figure 3F–3H). These results represented that Mark4 promoted VSMCs apoptosis and calcification probably through inhibiting the antiapoptotic effect of Akt signaling in β-GP-stimulated conditions.

### Sp1 participated in VSMCs apoptosis and calcification by increasing Mark4 levels

**Figure 4:**
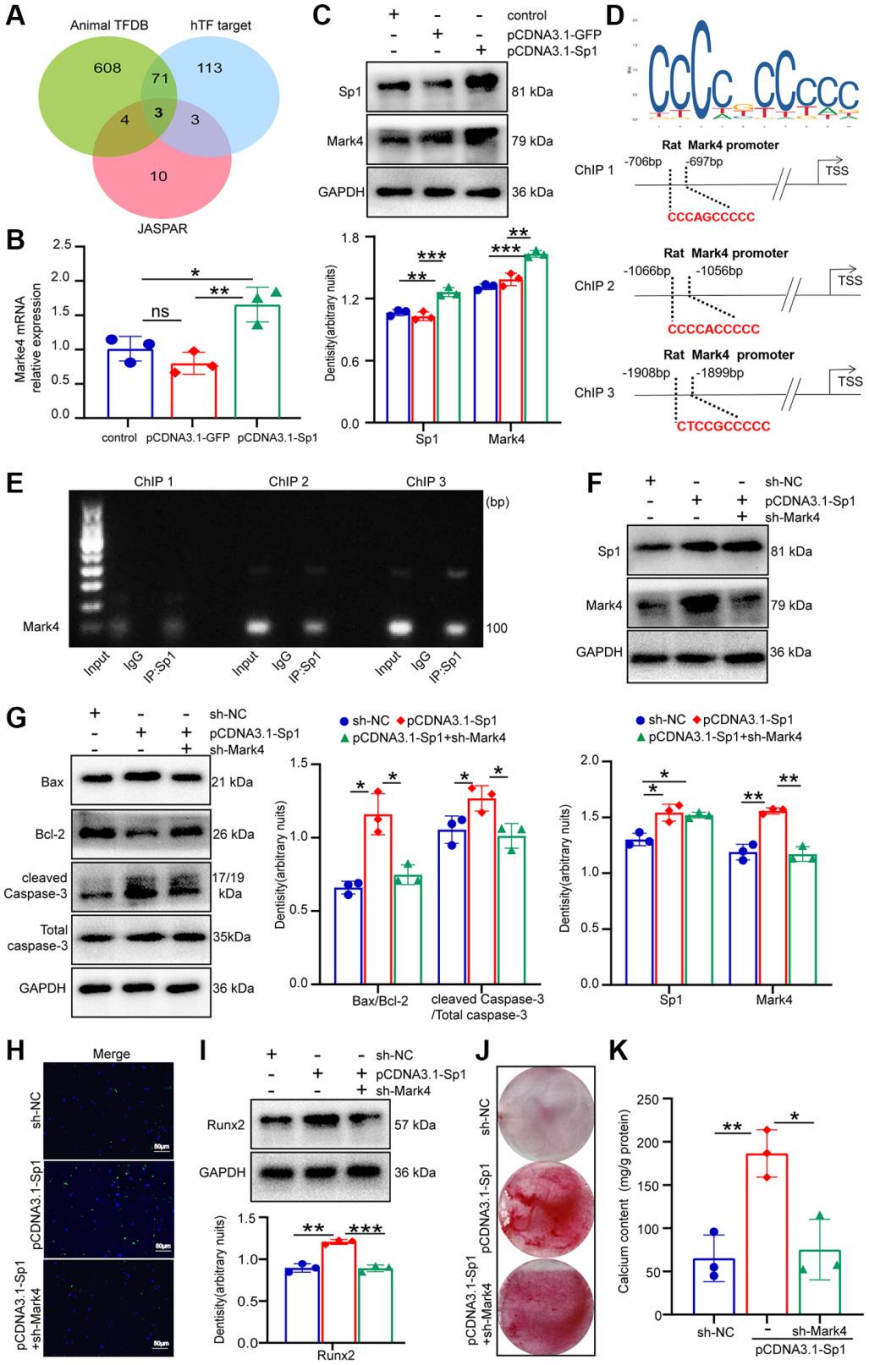
**Sp1 participated in β-GP-induced apoptotic protein levels and calcification via promoting Mark4 transcriptional activity in VSMCs.** VSMCs were infected with pCDNA3.1-Sp1 or its control pCDNA3.1-GFP and lentiviral shRNA Mark4 (sh-Mark4) or its negative control (sh-NC) under normal medium. (**A**) Three transcription factors, including Sp1, directly targeting the Mark4 were screened using the online bioinformatics databases Animal TFDB and hTF target, as well as JASPAR. (**B**) RT–qPCR analysis of Mark4 expression in pCDNA3.1-Sp1 transfected VSMCs. (**C**) Western blot and quantitative densitometry analysis of Sp1 and Mark4 in VSMCs with pCDNA3.1-Sp1. ^*^Means *p* < 0.05, ^**^p < 0.01, ^***^p < 0.001 vs. control. ^*^p < 0.05, ^**^p < 0.01, ^***^p < 0.001 vs pCDNA3.1-GFP. (**D**) The putative Sp1 binding site and sequences in Mark4 promoter were shown. (**E**) ChIP assay verified that Sp1 was enriched at the Mark4 promoter region. (**F**, **G**) Western blot and quantitative densitometry analysis of Sp1, Mark4 and apoptosis-related proteins in VSMCs with pCDNA3.1-Sp1 and sh-Mark4. (**H**) Apoptosis of VSMCs with pCDNA3.1-Sp1 and sh-Mark4 was assessed by TUNEL staining. (**I**) Western blot and quantitative densitometry analysis of Runx2, (**J**) Alizarin Red S staining and (**K**) calcium content assessed calcification in VSMCs with pCDNA3.1-Sp1 and sh-Mark4. ^*^Means *p* < 0.05, ^**^p < 0.01, ^***^p < 0.001 vs sh-NC, ^*^p < 0.05, ^**^p < 0.01, ^***^p < 0.001 vs. pCDNA3.1-Sp1. Error bar represents three independent experiments.

To uncover the potential molecular mechanism of Mark4 regulation in VSMCs calcification, bioinformatics (AnimalTFDB4, https://guolab.wchscu.cn/AnimalTFDB4/#/, hTFtarget, http://bioinfo.life.hust.edu.cn/hTFtarget#!/ and JASPAR, https://jaspar.genereg.net) were employed to predict the transcription factors that regulated Mark4 expression (Figure 4A). Combined with the predicted results, the Sp1, Glucocorticoid Receptor (NR3C1) and Estrogen Receptor 1 (ESR1) genes were highly associated with the expression of Mark4. Considering that Sp1 has been reported to play a vital role in the pathogenesis of VC [31], we focused on Sp1 as a potential molecule targeting Mark4 transcription. To investigate whether Sp1 was involved in modulating Mark4 levels, we carried out gain-of-function approaches. Overexpression of Sp1 enhanced Mark4 mRNA and protein expression, as shown in Figure 4B, 4C. Then, we sought to explore the molecular mechanism by which Sp1 modulated Mak4 transcription. Bioinformatics screening demonstrated that Sp1 might target three binding sites (marked as ChIP 1, 2, and 3) in the Mark4 promoter region (Figure 4D). ChIP-PCR assay validated that the enrichment of Sp1 detected within ChIP 3 (from-1908 bp to-1899 bp) was markedly stronger than that detected within ChIP1 and 2 (Figure 4E), further indicating that Sp1 amplified Mark4 expression via transcriptional activation. Subsequently, a further rescue experiment was conducted to define whether Sp1 facilitated apoptosis and calcification of VSMCs through promoting Mark4 expression. We downregulated Mark4 and simultaneously overexpressed Sp1, revealing that sh-Mark4 neutralized the effect of Sp1 overexpression on VSMCs apoptosis (Figure 4F–4H). Meanwhile, Mark4 silencing antagonized the increase in Runx2 levels and calcium nodule formation caused by Sp1 elevation (Figure 4I–4K). Collectively, these results demonstrated that Sp1 upregulation elevated Mark4 expression through transcriptional activation, then participating in the process of apoptosis and calcification in high phosphate-mediated VSMCs.

### Sp1 interacted with Setd8

**Figure 5:**
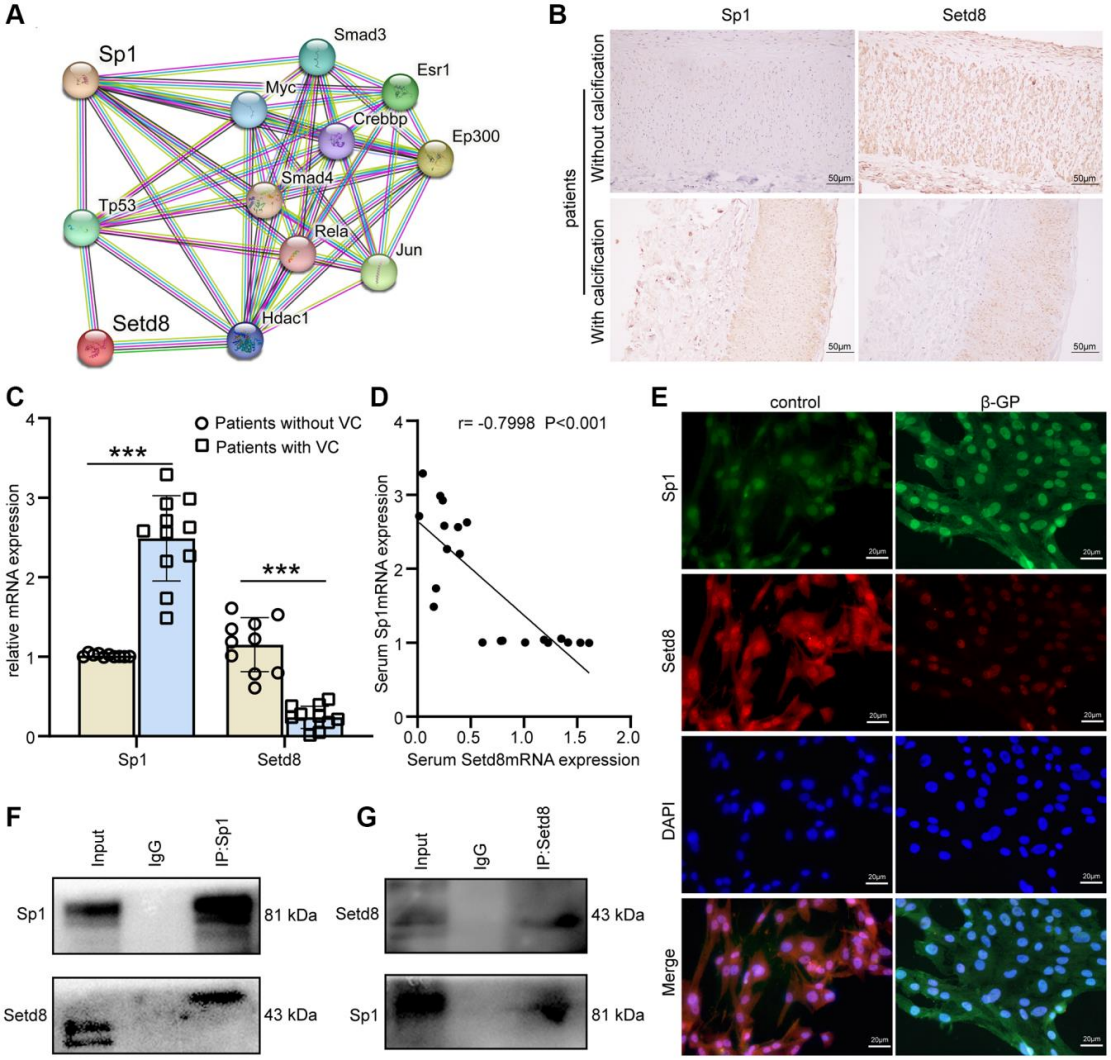
**Sp1 interacted with Setd8 to regulate Mark4 expression in VSMCs.** (**A**) Several proteins that interacted with Sp1 were shown (https://string-db.org). (**B**) Immunohistochemistry of Sp1 and Setd8 in CKD patients with or without arterial calcification. Scale bars: 50 μm. (**C**) RT–qPCR analysis of mRNA expression of Sp1 and Setd8 in serum from CKD patients with (*n* = 11) or without VC (*n* = 10). ^***^Means *p* < 0.001 vs. patients without VC group. (**D**) Correlation analysis between the expression of Setd8 mRNA and Sp1 mRNA in CKD patients (*n* = 21, the Pearson’s correlation coefficient r value and the *p*-value are shown). (**E**) Representative IF images showing the colocalization of Sp1 and Setd8. Scale bars: 20 μm. (**F**, **G**) The association between Sp1 and Setd8 in VSMCs was confirmed by Co-IP.

Bioinformatics (STRING, https://string-db.org) was used to predict the proteins interacting with Sp1 to reveal the underlying mechanism by which Sp1 regulated Mark4 expression (Figure 5A).

In the predicted results, Setd8 has been demonstrated to be responsible for VSMCs calcification, which is consistent with our previous studies; thus, we hypothesized that Setd8 interacted with Sp1. Initially, immunohistochemistry assay verified that Sp1 was increased and Setd8 was decreased in the aortic tissues of CKD-VC patients (Figure 5B). Similarly, elevated Sp1 mRNA and reduced Setd8 mRNA levels were observed in the serum of patients with CKD-VC (Figure 5C). Moreover, correlation analysis showed that serum Sp1 mRNA was significantly correlated with Setd8 mRNA (Figure 5D), suggesting a strong relationship between them. To further clarify the association of Sp1 and Setd8, we operated immunofluorescence double-staining assay. As expected, Setd8 co-localized with Sp1 in the nucleus of VSMCs treated with β-GP, manifesting as decreased red fluorescence intensity (Setd8) and increased green fluorescence intensity (Sp1) compared with that in the control group (Figure 5E). In addition, co-immunoprecipitation assays further confirmed that Setd8 and Sp1 have a reciprocal interaction in VSMCs (Figure 5F, 5G). Taken together, these data suggested that Setd8 interacted with Sp1 to participate in the modulation of Mark4 expression.

### Setd8 was involved in VSMCs apoptosis by regulating Mark4 expression under high phosphate conditions

**Figure 6:**
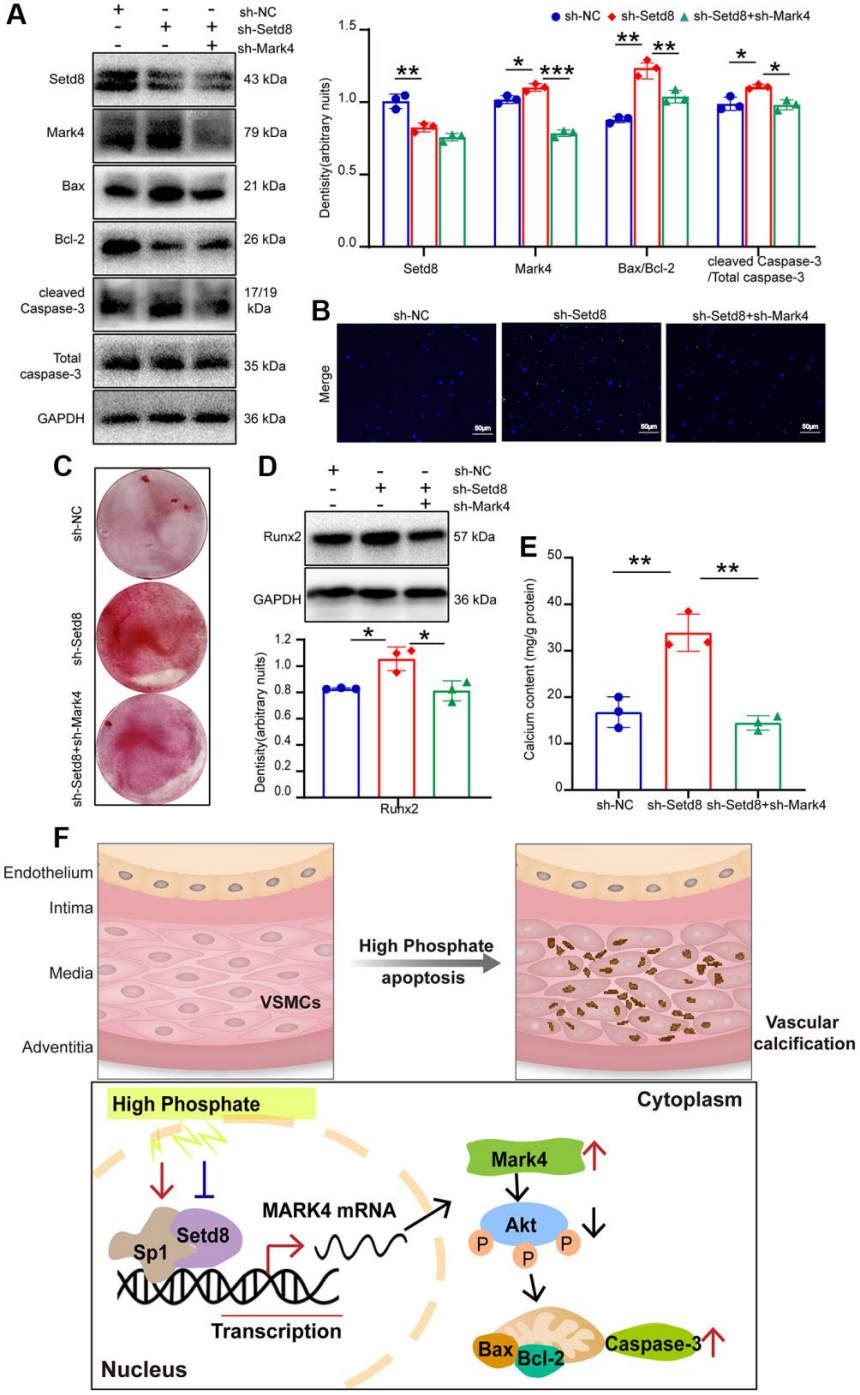
**Setd8 negatively regulated VSMCs apoptosis and calcification through augmenting Mark4 expression in VSMCs.** VSMCs were infected with lentiviral shRNA Setd8 (sh-Setd8) and shRNA Mark4 (sh-Mark4) or its negative control (sh-NC) in normal medium for 72 h. (**A**) Western blot analysis of Setd8, Mark4 and apoptosis-related proteins in VSMCs with sh-Setd8 and sh-Mark4. (**B**) Apoptosis of VSMCs with sh-Setd8 and sh-Mark4 was assessed by TUNEL staining. (**C**) Alizarin Red S staining in VSMCs infected with sh-Setd8 and sh-Mark4. (**D**) Western blot and quantitative densitometry analysis of Runx2. (**E**) Calcium content assessed calcification in VSMCs with sh-Setd8 and sh-Mark4. ^*^Means *p* < 0.05, ^**^p < 0.01, ^***^p < 0.001 vs. sh-NC, ^*^p < 0.05, ^**^p < 0.01, ^***^p < 0.001 vs. sh-Setd8. Error bar represents three independent experiments. (**F**) Schematic representation of the working model.

We next used sh-Mark4 in VSMCs with Setd8 downregulated to investigate whether the effects of Setd8 silencing arose from increasing Mark4 levels. Mark4 deficiency could partially reverse the proapoptotic effect of Setd8 inhibition, augmenting the expression of anti-apoptosis protein Bcl-2 and reducing the expression of proapoptotic proteins Bax and caspase-3 (Figure 6A). TUNEL assay further verified that Mark4 downregulation counteracted Setd8 silencing-induced cell apoptosis (Figure 6B). Furthermore, sh-Mark4 antagonized the Runx2 level increase caused by Setd8 silencing (Figure 6D). Consistently, knocking down Mark4 reduced calcium nodule formation and calcium deposition compared with that observed in the sh-Setd8 group, as determined by alizarin red staining and calcium content quantification (Figure 6C, 6E). These data revealed that Setd8 downregulation augmented apoptosis and calcification in VSMCs via upregulating Mark4 expression.

In summary, our results demonstrated that Sp1 could interact with Set8 to coactivate the transcription of Mark4, thereby inhibiting Akt phosphorylation and ultimately promoting VSMCs apoptosis and calcification under high phosphate conditions (Figure 6F).

## DISCUSSION

In the current study, Mark4 displayed a substantial increase in calcified models *in vivo* and *in vitro*, and promoted calcification progression by inhibiting Akt-mediated antiapoptotic effects. In addition, mechanistic studies showed that Sp1 interacted with Setd8 to modulate Mark4 expression by targeting a specific binding site (from −1908 bp to −1899 bp) in the Mark4 promoter region. These findings provide an innovative insight into the pathogenesis of VC and a potential new target for treating VC in patients with CKD.

Marks are a family of microtubule affinity-regulated kinases, involved in multiple physiological and pathological processes in various cells, such as hepatocytes, lipocytes and neurons [32–35]. The Marks contain four members of Mark1-4, which are located on human chromosomes 1, 11, 14 and 19, respectively, and play distinct physiological regulatory [36]. Numerous studies have shown that Mark4 is closely associated with the occurrence of diverse diseases, including cancer [13], Alzheimer’s disease [37], and cardiovascular disease [38, 39]. In the current study, we found that the expression levels of all Marks except Mark3 was increased in high phosphorus-induced VSMCs, while the elevation of Mark4 was the most pronounced. Again, high expression levels of Mark4 were detected in the serum and arterial tissues of CKD patients and rats with calcification, which was consistent with previous studies that Mark4 was elevated in the peripheral blood and aortic tissues of atherosclerotic humans and rats [40, 41]. Moreover, correlation analysis suggested a significant positive relationship between Mark4 expression and the degree of calcification. Thus, these findings indicated that Mark4 might be involved in the process of VC.

Mark4 is mainly located near the nucleus and is implicated in various cellular physiological functions through phosphorylating signaling pathway-related proteins or microtubule-associated proteins [42–44]. Previous evidence had shown that Mark4 knockdown suppressed NOD-like receptor protein 3-mediated inflammasome activation and pyroptosis in bone marrow-derived macrophages [45]. In addition, Mark4 promoted oxidative stress and mitochondrial dysfunction by activating NF-κB and inhibiting AMPK pathways in obesity-associated disorders [46]. Recently, Mark4 was reported to regulate cardiomyocyte contractility by promoting the phosphorylation of microtubule-associated protein 4 and acted as a promising therapeutic target for improving cardiac function after myocardial infarction [47]. In this study, we found that Mark4 aggravated VSMCs apoptosis and calcification *in vitro* VC models. Furthermore, in line with the results reported by Sun and Yang et al. [15, 48], silencing Mark4 reversed the inhibitory effect of high phosphorus on Akt, and promoting the expression of Akt phosphorylation protein had been indicated to inhibit apoptosis in VSMCs and attenuate VC [49, 50]. Overall, Mark4 could potentially aggravate VC by inhibiting the antiapoptotic role of Akt in VSMCs.

Sp1 is widely expressed and is the most active transcriptional activator, with at least 12,000 DNA binding sites in the human genome [22]. Sp1 can promote gene transcription, modulate the activity of encoded proteins, and is implicated in cardiac myocyte inflammation, apoptosis and fibrosis [51, 52]. *In vivo* and *in vitro*, we experimentally decerped that Sp1 expression was elevated in the arterial tissues of CKD-VC patients, increasing apoptosis and calcification which were consistent with previous studies [53, 54]. Zhang et al. recently showed that Sp1 could activate bone morphogenetic protein-2 transcription by binding to its promoter region to induce the phenotypic transformation of VSMCs or accelerate apoptosis-mediated VC [55]. Likewise, we first employed bioinformatics techniques to predict that Sp1 might act as a transcription factor regulating Mark4 expression. Then, Sp1 was proved to occupy the Mark4 promoter region at a specific binding site (from –1908 bp to –1899 bp), thereby evoking Mark4 transcription. Finally, knocking down Mark4 antagonized Sp1-mediated apoptosis and calcification. Based on these findings, we exhibited that Sp1 modulated the apoptosis of VSMCs and promoted the development and progression of VC by reinforcing Mark4 transcriptional activity.

Typically, Setd8 is known for monomethylating the lysine residues of histone, accelerating chromosome condensation, blocking transcription factors binding to the promoter region of target genes, and then repressing transcription of the genes [56–58]. Recent studies indicated that Setd8 could modulate the transcriptional activity of several transcription factors by epigenetic ways and subsequently supervise target gene expression [59, 60]. Qi et al. found that under hyperglycemia, Setd8 regulates Sp1 transcriptional activity, leading to an increase in target gene expression [61]. Similarly, our data demonstrated that Setd8 interacted with Sp1, co-localized in VSMCs nucleus and both enriched in the promoter region of Mark4, regulating Mark4 expression. In addition, Setd8 has been recognized as a potential effective target for cardiovascular disease and exerts its protective effects through mediating apoptosis in endothelial cells and smooth muscle cells, which is consistent with our previous reports [29, 61]. However, the regulatory mechanism of Setd8 in apoptosis has not been further elucidated. Our data corroborated that Setd8 attenuated VSMCs apoptosis by repressing Mark4 expression under high phosphate conditions.

In the present study, the upregulation of Mark4 in high phosphorus-stimulated VSMCs contributed to cell apoptosis and calcification via inhibiting anti-apoptotic effect of Akt. Further investigations verified that Setd8 can directly interact with Sp1 and enhance Sp1-mediated Mark4 transcriptional activation, ultimately upregulating Mark4. Finally, our data for the first time supplied a new insight into the role of Mark4 in VSMCs apoptosis and calcification, and provided a potential therapeutic target for VC in patients with CKD.

## MATERIALS AND METHODS

### CKD patient recruitment

This case-control study included patients hospitalized at the Fourth Hospital of Hebei Medical University from June 2022 to August 2022. It was approved by the Ethics Committee of the Fourth Hospital of Hebei Medical University (No. 2022KY033). Informed consent was taken from all participants. A total of 21 CKD patients with estimated glomerular filtration rate (eGFR) <15 mL/min/1.73 m^2^ were included in this study. Exclusion criteria: diabetes mellitus, malignancy, severe malnutrition, uncontrolled hyperlipidemia, acute cardiovascular events, acute infections, chronic active hepatitis, fractures occurring in the past 6 months, or patients older than 80 years or younger than 18 years.

### CKD patient samples

Fasting elbow venous blood was collected, centrifuged at 3000 g for 10 min, and the supernatant was removed and subsequently stored at −80°C for total RNA extraction. Radial artery segments of 3–5 mm were obtained from CKD patients who underwent arteriovenous stoma prior to hemodialysis, and were placed in 4% paraformaldehyde after removal of fat and outer membrane, followed by paraffin embedding.

### Calculation of calcification score

According to the criteria, Angaston scores were calculated by three independent image analysts blinded using Smart Score software (Definition Flash, Siemens Healthcare, Florsheim, Germany) [62]. Patients were classified as non-VC (Angaston score = 0) and with-VC (Angaston score >0) according to coronary artery calcification score criteria.

### Serum RNA extraction and reverse transcription

The serum RNA samples were extracted using TRIzol reagent (Invitrogen, Carlsbad, CA, USA, 15596026). The concentration of total RNA was measured by a Visible Spectrophotometer (EMCLAB, Duisburg, Germany). Reverse transcription was then performed using a Supersmart 6 min 1st Strand cDNA Synthesis Kit (Zsgentech, Tianjing, China, ZS-M1092) to obtain complementary DNA (cDNA). A SYBR Green I-based real-time quantitative PCR was conducted to analyze the expression of target gene. The relative mRNA level was determined using the comparative CT method and was normalized to the housekeeping gene glyceraldehyde-3-phosphate dehydrogenase (GAPDH). The primers sequences were shown in Supplementary Table 1 and synthesized by the Sangon Biotech (Shanghai, China).

### Animal models

Sprague-Dawley (SD) rats (aged 8-weeks) weighing 180 to 220 grams were purchased from the Lab Animal Center of Hebei Medical University (Hebei Province, China). Male rats were selected to exclude the potential interference of estrogen on VC. All rats were kept in temperature- and humidity-controlled conditions on a 12-hour dark/light cycle with accessible chow and tap water. The rats were divided into the control and VC groups using a simple random sampling method. The control group was fed a standard normal chow diet, while the VC group was induced for 6 weeks with a special chow containing 0.75% adenine and high phosphorus levels (1.2%). Our procedures in animal experiments were performed under the guidance of the experimental Animal Welfare Ethics Committee.

### Cell culture and calcification induction

Human arterial smooth muscle cells (ScienCell, Carlsbad, CA, USA, no. 6110) were cultured in smooth muscle cell medium containing apo-transferrin, insulin, fibroblast growth factor-2, insulin-like growth factor-1, hydrocortisone, and 2% fetal bovine serum (ScienCell, Carlsbad, CA, USA, no. 1101).

Rat VSMCs was extracted from healthy male SD rats aged 8 weeks. After isoflurane anesthesia, the rat thoracic aortas were rapidly excised, the outer and inner membranes of the vessels were completely removed, and the arterial segments were cut into sections and then immersed in DMEM supplemented with 10% FBS. After suffusion, tissue debris was removed, and cells between the 3rd and 5th generation were used for the subsequent experiments. Mycoplasma detection was performed on the cells using the Mycoplasma alarm plus Mycoplasma detection kit (Lonza, Basel, Switzerland) according to the manufacturer’s instructions.

Cells were cultured in growth medium in the presence of β-glycerophosphate (β-GP) (10 mM, Aladdin, Shanghai, China) for 14 days to induce calcification. The medium was refreshed every 2 days. All experiments with VSMCs were performed using cells from independent batches.

### Determination of calcification

Five-micrometer paraffin sections of the arteries were used to detect aortic calcification by Von Kossa staining. The sections were dehydrated and incubated with 5% silver nitrate solution under ultraviolet light for 1 h, and the calcium nodules were stained brown.

For Alizarin red staining, the calcified cells were fixed with 4% formaldehyde for 30 min and then stained with 1% Alizarin red-Tris-HCL solution pH 4.2 (Solarbio Technology Co., Beijing, China, G1452) at room temperature for 15 min. Positively stained cells were red-orange in color, indicating calcification.

For the calcium content assay, cells were washed three times with phosphate buffered saline (PBS), incubated overnight with 0.6 mol/L hydrochloric acid, and the cell supernatant was then collected. Released calcium was measured using the Calcium Colorimetric Assay Kit (Beyotime Biotechnology, Shanghai, China, S1063S) as per in the manufacturer’s protocol. The calcium levels were normalized to the total protein concentration.

### Western blot

After extraction from VSMCs with lysis buffer, equal amounts of protein per lane were loaded onto 10% or 12% SDS-polyacrylamide gels. After electrophoresis and electro-transfer to polyvinylidene difluoride membranes, the gels were closed with 5% BSA (BioFroxx, Guangzhou, China, 4240GR100) for 1 h at 37°C and then incubated with primary antibodies at 4°C overnight. After washing with 1% Tween-Tris buffered saline, the membranes were incubated with the secondary antibodies at room temperature for 1 h. Then, the protein signal was detected using an ECL system (Thermo Fisher Scientific, Waltham, MA, USA, 32106) and a Fluor Chem R imaging system (Protein Simple, San Jose, CA, USA). For quantitative analysis, band intensity was quantified using ImageJ software and the relative density was the ratio of the target gene to GAPDH. The antibody details can be found in Supplementary Table 2.

### Realtime-quantitative polymerase chain reaction (RT-qPCR)

Total RNA was extracted from VSMCs using TRIzol reagent (Invitrogen, Carlsbad, CA, USA, 15596026) according to the manufacturer’s instructions. For mRNA quantification, a Supersmart 6 min 1st Strand cDNA Synthesis Kit (Zsgentech, Tianjing, China, ZS-M1092) was used for RNA reverse transcription into cDNA. Real-time fluorescent quantitative PCR amplification was performed using Superbrilliant5 × Fast SYBR Green qPCR Mix (Zsgentech, Tianjing, China, ZS-M13001). Relative quantification was performed using the 2^−ΔΔCt^ method and normalized by GAPDH expression. The primer sequences are shown in Supplementary Table 1. The experiments were repeated three times for each gene.

### Plasmids and cell transfection

The small hairpin RNA against Mark4 (sh-Mark4), Setd8 and negative control shRNA were obtained from Gene Pharma Biotechnology Co. Ltd. (Shanghai, China). The plasmid sh-Mark4 has a green fluorescent label (pGPU6/GFP/Neo) and if successfully transfected, green fluorescence was visualized with light at 488 wavelengths. The sequences of sh-Mark4 were shRNA-a 5′GGACACGGAGCTCAAAGAAGA3′, shRNA-b 5′GCCAGTCCTTGTTGCCAAATG3′ and shRNA-c 5′GGGCTACACACGGGAAGAAAT3′. The sequences of shSetd8 (sh-Setd8) (Gene Pharma Biotechnology Co., Ltd., Shanghai, China) were 5′CCGACAACCACTACCTGA3′. The pCDNA3.1-Sp1 and corresponding empty plasmids were constructed by Gene Chem Company (Shanghai, China). VSMCs were transfected with different plasmids using Lipofectamine 3000 (Invitrogen Carlsbad, CA, USA, L3000015) according to the manufacturer’s instructions.

### Immunofluorescence

The VSMCs were fixed with 4% paraformaldehyde, followed by 0.3% TritonX-100 to increase the permeability of the cell membrane. After being closed with goat serum for 1 hour, cells were incubated with primary antibodies against Sp1 and Setd8 at 4°C overnight.

Artery sections were deparaffinized in xylene and rehydrated through a graded alcohol series to distilled water. Antigen retrieval was performed by microwave irradiation in EDTA. Then tissue sections were incubated with 1% normal goat serum for 1 hour to reduce nonspecific background staining. Sections were then incubated overnight at 4°C with primary antibody for Mark4 and Runx2.

Finally, cells and arteries were observed after incubation with secondary antibody and DAPI (Solarbio Technology Co., Beijing, China, S2110). The antibody details can be found in Supplementary Table 2.

### Immunohistochemistry

Paraffin-embedded sections of artery tissues were permeabilized and dehydrated. Sections were then incubated with 3% hydrogen peroxide to burst endogenous peroxidase, and goat serum was used to block nonspecific staining. Next, the incubation of sections was stained with primary antibodies against Mark4, Sp1, Caspase-3, Runx2 and Setd8 at 4°C overnight, followed by biotin-coupled secondary antibody (ZSGB-BIO, Beijing, China, PV-9000) staining for 40 min at 37°C. Finally, sections were covered with DAB to show positive staining. The images were collected under an Olympus microscope. The antibody details can be found in Supplementary Table 2.

### Co-immunoprecipitation (Co-IP)

Live cell proteins were extracted with cell lysis buffer containing PMSF (Solarbio Technology Co., Beijing, China, P0100) lysate. For endogenous IP, 30 μl of lysate was incubated with the corresponding primary antibody and 50 μl of protein A/G beads (Geneseed Biotech Co., Guangzhou, China, P0102) for 12 h at 4°C. Then, 10 μl of the input, IgG, and IP fractions were subjected to Western blot.

### Chromatin immunoprecipitation (ChIP) analysis

ChIP assay was performed by using an EpiQuik (ChIP) Kit (Epigentek, NY, USA, P-2002) according to the manufacturer’s instructions. First, the antibodies against Sp1 and negative control IgG were added to the corresponding strip wells and incubated at room temperature for 90 min. Second, cells (1 × 10^7^) were fixed with 1% formaldehyde for 10 min at room temperature to cross-link DNA and proteins. The cross-linking reaction was terminated by glycine, and the DNA-protein binding product was sheared by an ultrasonic cell disruptor (Kun Shan Ultrasonic Instruments Co., Ltd., Jiangsu, China, KBS-150). Third, 5 μl of the sonication solution was withdrawn as the input, while the remaining solution was transferred to strip wells and incubated at room temperature (22–25°C) for 60–90 min on an orbital shaker (50–100 rpm). Finally, after performing purification of the DNA-protein crossover, the enriched DNA sequences were analyzed by PCR. The Mark4 oligonucleotide primer sequences are listed in Supplementary Table 3.

### TUNEL assay

The number of apoptotic VSMCs was quantified using a terminal deoxynucleotidyl transferase-mediated dUTP nicked labeling (TUNEL) kit (Meilunbio, Shanghai, China, MA0223) according to the manufacturer’s instructions. Briefly, VSMCs were fixed with 4% paraformaldehyde and punched with 0.2% Triton-100 for 1 h. Subsequently, the cells were incubated with the TUNEL mixture for 1 h and viewed using a fluorescence microscope.

### Statistical analysis

For all experiments, three technical replicates were performed. The results are expressed as the mean ± standard deviation (SD). Analysis was performed using SPSS 17.0 software; *t*-tests or one-way analysis of variance (ANOVA) were used to assess the significance of differences between two groups or among multiple sample groups. Pearson correlation analysis was used to assess the correlation between the two variables. The two-tailed *P*-value < 0.05 was considered as significant differences.

### Data availability statement

The datasets used to support the findings of the current study are available from the corresponding author upon reasonable request.

## Supplementary Materials

Supplementary Tables
