# Supplementary material for: Interaction of Sp1 and Setd8 promotes vascular smooth muscle cells apoptosis by activating Mark4 in vascular calcification
Source: Aging (Albany NY). 2024 Feb 1;16(3):2438–56. doi: 10.18632/aging.205492 (PMC10911351; doi:10.18632/aging.205492)
Supplement: Supplementary Tables [file aging-16-205492-s001.pdf]

SUPPLEMENTARY TABLES

Supplementary Table 1. The sequences of primers used for RT-qPCR.

Gene	Forward primer	Reverse primer
	**Human**	
Mark1	GAGCGGGACACGGAAAATCAT	TGCTACTCGACTTGGTAGGCT
Mark2	CACATTGGAAACTACCGGCTC	GGAGGAGTTCAGTTGAGTCTTGT
Mark3	GGAGGAGTTCAGTTGAGTCTTGT	GCCTGTAAGGATATGTCTTGCC
Mark4	AGGTTGCCATCAAGATTATCGAC	GATGCGGACTTCTCGGAACAG
Sp1	AGTTCCAGACCGTTGATGGG	GTTTGCACCTGGTATGATCTGT
Setd8	TCCAGCAATCCTCCTCCTTCCTC	CCAGCCTAAGCAACAGATCCAGA
	**Rat**	
Mark4 Setd8	TGATGACCCGAAGAAACACCT<br>AAAGACGCCAGGAAAGGTCC	GCTGTGACTGGAGGGTGAG<br>TTCCACCACAAAGTCACCCC

Supplementary Table 2. The antibodies used in this study.

Anti-Mark4	CST	#4834	WB 1:500; IHC/IF: 1:50
Anti-Caspase-3	CST	#9662	WB 1:1000; IHC: 1:200
Anti-phosphor-Akt (Ser473)	CST	#4060	WB 1:1000
Anti-Akt	CST	#9272	WB 1:1000
Anti-Sp1	Proteintech	#2196-1-AP	WB 1:1000; IHC/IF 1:200; IP: 5 µl for 1mg protein; Chip :4 ug
Anti-BAX	Abcam	#ab32503	WB 1:2000
Anti-Bcl2	Abcam	#ab196495	WB 1:1000
Anti-Runx2	Abcam	#ab76956	WB 1:500; IHC/IF 1:200;
Anti-Setd8	Santa	#sc377034	WB 1:500; IHC/IF 1:100; IP: 5 µl for 1mg protein
GAPDH	Bioworld Technology Co	#AP0063	WB 1:1000
Anti-rabbit	Report	#S1002	WB: 1:5000
Anti-mouse	Seracare	#S1001	WB: 1:1000
Anti-rabbit-DyLight488	Abbkine	#A23220	IF: 1:200
Anti-mouse-DyLight 594	Abbkine	#A23410	IF: 1:200
Rabbit control IgG	ABclonal	#AC005	IP: 3 µl, Chip:1 µl

Supplementary Table 3. The sequences of primers used for ChIP.

Gene	Forward primer	Reverse primer
Mark4-1	CTGGGATTTGAACTGAGGAC	CAAGCTGGTTATAGGACTGG
Mark4-2	CTTTCCTCCCACAAGGCACT	TAGCTGGAAGCCCAAACAGG
Mark4-3	TTCTTCCCACCTCGCGTCTG	CCGGCTCTTAAAGGCACAGG
